# The Electric Mouth Lamp

**Published:** 1884-12

**Authors:** 


THE ELECTRIC MOUTH LAMP OR STOMATOSCOPE.—This appliance has been for some time past in use in the Dental Infirmary of the University of Maryland, and is indeed an invaluable assistant in detecting a hidden cavity of decay on unsuspected dead pulp, or even a slight thickening of tissues, indeed any departure from normality. A mirror attached to the guard in front of the lamp enables the operator to examine posterior cavities in teeth. It can be readily operated by means of a current from a two or three cell Bunsen battery.

